# Duplex Ultrasound Surveillance After Endovascular Therapy for Peripheral Artery Disease: An Australian and New Zealand Study

**DOI:** 10.1111/ans.70329

**Published:** 2025-09-25

**Authors:** Thomas M. Warburton, Shannon D. Thomas, Manar Khashram, Peter Subramaniam, Fernando Picazo‐Pineda, Simon Joseph, Andrew F. Lennox, Nedal Katib, Ramon L. Varcoe

**Affiliations:** ^1^ Department of Surgery Prince of Wales Hospital Sydney Australia; ^2^ Faculty of Medicine University of New South Wales Sydney Australia; ^3^ The Vascular Institute Prince of Wales Hospital Sydney Australia; ^4^ Department of Vascular Surgery Waikato Hospital Hamilton New Zealand; ^5^ Faculty of Medicine and Health Sciences University of Auckland Auckland New Zealand; ^6^ Department of Vascular Surgery Royal Adelaide Hospital Adelaide Australia; ^7^ Faculty of Health and Medical Sciences University of Adelaide Adelaide Australia; ^8^ Department of Vascular Surgery Royal Perth Hospital Perth Australia; ^9^ Department of Vascular Surgery Sir Charles Gairdner Hospital Perth Australia

**Keywords:** ankle brachial index, duplex ultrasound, endovascular intervention, peripheral artery disease, surveillance

## Abstract

**Objective:**

Current practice guidelines lack consensus on optimal surveillance strategies following endovascular interventions for peripheral artery disease (PAD). This study evaluated surveillance practices among vascular surgeons in Australia and New Zealand (ANZ), focusing on duplex ultrasound (DUS) use and factors influencing surveillance protocols.

**Methods:**

All consultant vascular surgeons in the Australian and New Zealand Society for Vascular Surgery were invited to participate in an online survey examining demographics, practice characteristics, surveillance protocols after endovascular interventions, and decision‐making regarding surveillance and reintervention.

**Results:**

Of 266 surgeons, 73 responded (27%). Respondents were predominantly experienced (77% with ≥ 10 years practice) and worked in teaching hospitals (88%). Most performed an initial post‐procedural DUS (89%) and ongoing routine DUS surveillance (83%). Surveillance protocols varied considerably, with 67% tailoring intervals based on patient/lesion factors rather than predetermined schedules. Practice variation was independent of surgeon experience, practice setting, or geography. All surveyed chronic limb‐threatening ischemia patients, versus 93% for intermittent claudication. Reintervention thresholds varied by anatomy: 58% required symptoms before intervening for infrapopliteal disease compared to 15% for aortoiliac disease. Most surgeons (82%) acknowledged significant uncertainty regarding optimal surveillance strategies.

**Conclusion:**

Substantial practice variation exists in post‐endovascular surveillance among ANZ vascular surgeons. While most employ DUS surveillance, frequency, duration, and intervention thresholds differ markedly. These findings highlight the need for prospective studies to determine optimal surveillance protocols balancing clinical outcomes with resource utilization.

## 1 Introduction

Endovascular treatment of peripheral arterial disease (PAD) often experiences limited durability due to restenosis compromising long‐term vessel patency [1, 2].

Regular duplex ultrasound (DUS) surveillance, combined with clinical assessment, enables early detection of restenosis and timely reintervention [3, 4]. However, optimal post‐interventional surveillance strategies remain undefined, with significant variations in current practice guidelines [3, 5, 6].

The European Society for Vascular Surgery recommends periodic clinical evaluation with ankle‐brachial index measurement, offering only weak recommendations for DUS surveillance [7]. The Society for Vascular Surgery takes a more stratified approach, recommending clinical surveillance alone for intermittent claudication but intensive surveillance for chronic limb‐threatening ischemia [3]. The absence of evidence‐based consensus, combined with the acknowledgment by both societies that their recommendations are based on expert opinion rather than high‐quality comparative data, underscores the need for definitive randomized controlled trials to establish optimal surveillance strategies.

This uncertainty mirrors the more established evidence regarding surveillance after open surgical revascularization, where DUS has not demonstrated clear benefits in detecting stenosis and maintaining graft patency [8]. However, the applicability of these findings to endovascular interventions remains unclear, given the differing failure patterns and reintervention options for open and endovascular methods.

To better understand current practice patterns in Australia and New Zealand, we conducted a survey of vascular surgeons to characterize surveillance strategies after endovascular interventions for PAD. Our aim was to evaluate the extent of practice variation, identify factors influencing surveillance decisions, and assess interest in future research to establish evidence‐based guidelines.

## 2 Methods

### 2.1 Survey Development and Distribution

An online survey was developed to assess current practice patterns regarding surveillance after endovascular interventions for PAD, which is made available in the appendices. Demographic elements of the survey were designed for comparison with the recent Dutch survey of vascular surgical surveillance practices [9]. The survey was pilot tested with two senior vascular surgeons to ensure clarity and completeness before distribution. The final survey was distributed electronically via Qualtrics (Qualtrics, Provo, UT) to all consultant vascular surgeons (266) registered with the Australian and New Zealand Society for Vascular Surgery (ANZSVS) between March and May 2025, with two reminder emails. The ethical suitability of the survey was reviewed and approved by the ANZSVS Executive prior to distribution.

### 2.2 Survey Content

The survey included questions on:

1. Demographic information (years of practice experience, location, public vs. private practice setting).
2. Practice characteristics (types of facilities, endovascular capabilities).
3. Use of post‐procedural surveillance (initial and long‐term).
4. Surveillance modalities (clinical examination, ABI, DUS, CT angiography).
5. Factors influencing surveillance strategies (patient factors, lesion characteristics, indication for intervention).
6. Thresholds for reintervention in asymptomatic patients.
7. Interest and opinion on future randomized research on surveillance strategies.

The survey primarily used multiple‐choice questions with the option for free‐text responses where appropriate. Surveillance was defined as any post‐procedural follow‐up, including clinical examination with or without additional diagnostic testing.

### 2.3 Statistical Analysis of Practice Variations

Survey responses were collected anonymously. Descriptive statistics were used to summarize respondent characteristics and surveillance practices. To examine associations between surgeon characteristics and surveillance practices, univariate statistical analyses were performed. Chi‐squared tests were used to assess relationships between categorical variables when expected cell frequencies were adequate (≥ 5 per cell). When assumptions for chi‐squared tests were violated due to small expected cell counts, Fisher's exact tests were employed. For ordinal variables or when comparing groups with non‐normally distributed data, Kruskal–Wallis tests were used. Statistical significance was set at *p* < 0.05.

Multivariate analysis was not performed due to the limited sample size relative to the number of potential predictor variables, which would have risked model overfitting and unreliable estimates. All analyses were performed using Qualtrics' built‐in statistical software (Qualtrics, Provo, UT).

## 3 Results

### 3.1 Respondent Characteristics

The survey had a response rate of 73 out of 266 vascular surgeons (27%). Most respondents were extensively experienced vascular surgeons, with 40% having practiced for ≥ 20 years and 37% for 10–20 years (Table 1). The majority (88%) worked primarily in teaching hospitals, with the highest representation from New South Wales (27%), Victoria (22%), and New Zealand (19%).

**TABLE 1:** Demographic characteristics of survey respondents, including years of experience as a consultant vascular surgeon, hospital type, and geographical distribution.

Characteristic		Respondents, *n* (%)
Years of experience as a vascular surgeon		
< 5 years		12 (16%)
5–9 years		5 (7%)
10–19 years		27 (37%)
≥ 20 years		29 (40%)
Region of Australia or New Zealand		
New South Wales		20 (27%)
Victoria		16 (22%)
New Zealand		13 (19%)
Queensland		9 (12%)
Western Australia		8 (11%)
South Australia		3 (4%)
Tasmania		2 (3%)
Australian Capital Territory		1 (1%)
Northern Territory		0 (0%)
Hospital type		
Teaching hospital		64 (88%)
Metropolitan nonteaching hospital		6 (8%)
Regional nonteaching hospital		3 (4%)
Practice		
Surgeon performs endovascular therapy for PAD		71 (97%)
Portion of public and private surgical work	A mix of public and private	45 (62%)
	Chiefly public (little or no private)	20 (27%)
	Chiefly private (little or no public)	8 (11%)
Room available for endovascular interventions		
Hybrid theatre		53 (72%)
Interventional/radiology suite		48 (66%)
Operative theatre with mobile x‐ray		13 (18%)

Most surgeons (97%) reported performing endovascular interventions for lower limb PAD. Surgeons had access primarily to hybrid theatres (72%) and interventional radiology suites (66%), with some surgeons using conventional operating rooms with mobile x‐ray equipment (18%).

### 3.2 Surveillance Practices

Following lower limb endovascular intervention, 89% of surgeons performed an initial post‐procedure DUS, typically at 4–6 weeks post‐intervention. The majority of surgeons (83%) reported performing routine ongoing DUS surveillance after lower limb endovascular interventions, beyond the initial post‐procedural review. DUS surveillance is adjunct to physical examination at a minimum.

The duration of surveillance varied considerably among respondents. One‐third (33%) conducted lifelong surveillance, while 24% performed surveillance for 2 years, 12% for 1 year, and 10% for 5 years. The remaining 21% selected “other,” with many specifying variable durations based on patient factors such as age, comorbidities, and lesion complexity.

Regarding surveillance frequency, 67% reported that their schedule varied depending on patient or lesion factors. Fixed surveillance intervals were less common, with 14% performing DUS every 6 months, 10% annually, and 9% every 3 months.

### 3.3 Associations Between Surgeon Characteristics and Surveillance Practices

There were no statistically significant associations found between key surgeon characteristics (clinician experience, location, public vs. private practice setting) and surveillance practices (modality, regularity, or duration) across multiple comparisons.

There was no significant relationship between the proportion of public versus private surgical work and whether clinicians performed routine post‐procedural DUS surveillance (*p* = 0.732), nor surveillance regularity (*p* = 0.258) or duration of surveillance (*p* = 0.186). Years of consultant experience showed no significant association with performing routine post‐procedural DUS surveillance (*p* = 0.476), surveillance duration (*p* = 0.289), or regularity (*p* = 0.071). No significant regional differences were found in surveillance practices across Australia and New Zealand, including routine DUS surveillance performance (*p* = 0.511), surveillance regularity (*p* = 0.449), or surveillance duration (*p* = 0.569).

### 3.4 Surveillance by Anatomical Location

**FIGURE 1:**
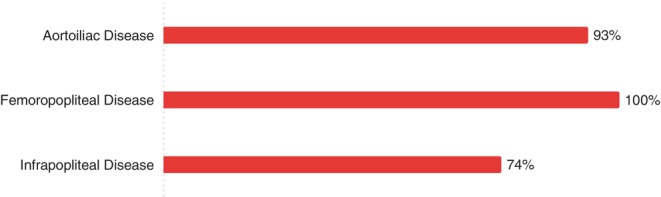
Surveillance rate by arterial segment.

Surveillance rates varied by anatomical location, with the femoropopliteal segment having the highest surveillance rate and infrapopliteal the lowest (Figure 1).

### 3.5 Surveillance by Clinical Indication

**FIGURE 2:**
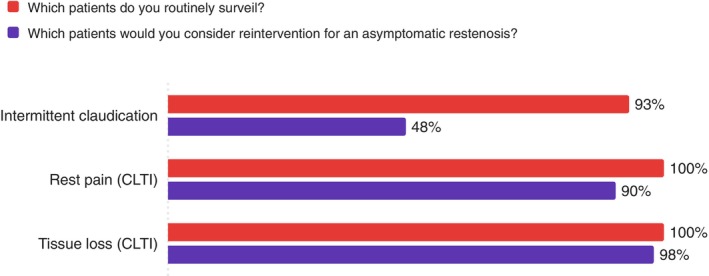
Surveillance and reintervention rates by clinical indication.

Surveillance patterns differed based on the indication for intervention. Surveillance and reintervention practices were stratified by clinical indication, with near‐universal surveillance for CLTI patients and more conservative approaches for intermittent claudication (Figure 2).

### 3.6 Surveillance by Intervention Type

**FIGURE 3:**
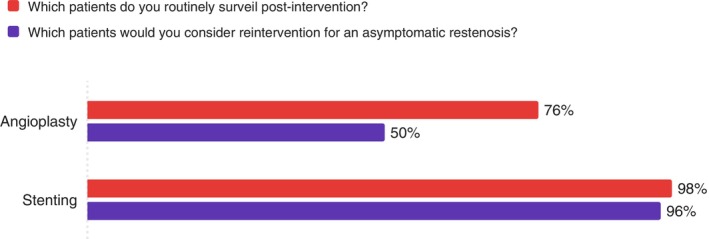
Surveillance and reintervention rates by intervention type.

Both surveillance rates and reintervention thresholds were higher following stent placement compared to angioplasty alone (Figure 3).

### 3.7 Thresholds for Reintervention by Anatomical Location

**FIGURE 4:**
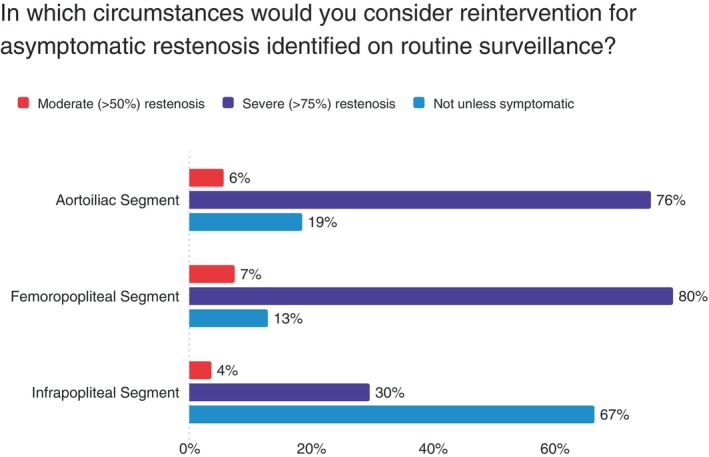
Threshold for reintervention for asymptomatic restenosis by anatomical location.

Reintervention thresholds demonstrated a clear anatomical hierarchy, with surgeons most willing to intervene for proximal disease and severe stenosis across all segments (Figure 4).

### 3.8 Clinical Equipoise and Research Interest

When asked about clinical equipoise regarding optimal surveillance strategies, 82% agreed that there was uncertainty in the benefit of DUS surveillance after endovascular therapy for PAD. The majority (63%) expressed interest in participating in a trial comparing different surveillance strategies, with 24% indicating potential interest and 13% declining.

Almost all respondents (95%) reported having the capacity to perform clinical reviews, ABI measurements, and vascular ultrasounds in their units. Regarding potential randomized trials, 55% stated that the randomization would not influence their willingness to enroll patients; however, a significant portion (43%) of respondents favored a 2:1 randomization scheme, which would have a higher chance of any given patient falling into the DUS arm.

## 4 Discussion

This survey reveals that vascular surgeons in Australia and New Zealand are practicing in an environment of considerable uncertainty in surveillance after endovascular interventions in PAD patients. While the majority use DUS surveillance (83%), there is no consensus on how often to perform it, how long to continue it, or what findings should trigger further intervention. The variation in practice appears to be driven by practical considerations. Surgeons are more aggressive with surveillance and reintervention when critical limb‐threatening ischemia is the presenting complaint, and when the technical complexity of dealing with failure is greater (such as after stenting vs. angioplasty alone) [10]. Statistical analysis found insufficient evidence that practice variations were associated with surgeon experience, practice setting (public vs. private), or geographic location. Most tellingly, over 80% of surgeons acknowledge significant uncertainty about optimal surveillance strategies, indicating they are practicing without clear evidence‐based guidance.

Our findings align closely with a Dutch survey by Hoitz et al., which similarly demonstrated substantial heterogeneity in surveillance practices among vascular surgeons [9]. However, our study found a higher prevalence of routine DUS surveillance (83% vs. 21%–60% in the Dutch study). This difference likely reflects regional healthcare system variations, with Australia and New Zealand's mixed public and private healthcare infrastructures potentially providing greater access to diagnostic imaging compared to the Netherlands. Both studies identified similar patterns of increased surveillance intensity for higher‐risk patients and anatomical locations, suggesting these clinical decision‐making patterns are likely common internationally.

We observed an anatomical hierarchy, with nearly universal surveillance of aortoiliac and femoropopliteal interventions but reduced surveillance of infrapopliteal interventions (74%), mirroring findings from systematic reviews of surveillance literature [4]. This pattern likely reflects both the technical challenges of monitoring smaller vessels and the variable clinical implications of recurrent infrapopliteal disease. Our finding that surgeons are more likely to surveil and reintervene after stenting compared to angioplasty alone also aligns with established clinical reasoning, as stent occlusion typically presents more complex management challenges than restenosis after balloon angioplasty [11].

An important finding from our statistical analysis was the absence of significant associations between surgeon characteristics and surveillance practices. Neither years of experience, practice setting (public vs. private), nor geographic location influenced whether surgeons performed routine surveillance or their chosen surveillance protocols. This lack of association suggests that practice variation is not driven by systematic differences in training, resource availability, or regional practice cultures, but rather reflects the genuine uncertainty that exists in the absence of evidence‐based guidelines. The fact that both junior and senior surgeons, those in public and private practice, and clinicians across different states and countries all demonstrate similar patterns of practice variation underscores that this uncertainty is pervasive across the specialty. While we observed substantial descriptive variation in surveillance practices, our statistical analysis focused on associations between surgeon characteristics and practice patterns rather than formal testing of practice heterogeneity.

This survey was undertaken as preparatory work for the forthcoming SURVEIL randomized controlled trial, which aims to compare DUS surveillance with clinical surveillance alone following endovascular intervention for peripheral arterial disease. The SURVEIL trial is registered as ACTRN12625000889459p (prospectively registered) under the Australian New Zealand Clinical Trial Registry.

Several limitations should be acknowledged in interpreting these findings. The 27% response rate, while reasonable for physician surveys, may not fully represent the practices of all Australian and New Zealand vascular surgeons, potentially introducing selection bias toward more engaged practitioners. Self‐reported practices may differ from actual clinical behavior, though this limitation is inherent to survey methodology. The survey focused on current practices without exploring the detailed clinical reasoning behind individual decisions, which limits our understanding of why such variation exists. Statistical analysis was limited to univariate comparisons due to sample size constraints that precluded reliable multivariate modeling. This prevents assessment of potential confounding or interaction effects between surgeon characteristics. We also did not address cost‐effectiveness considerations or resource utilization patterns, which are increasingly important factors in clinical decision‐making. We did not assess whether clinicians had access to in‐hospital public laboratories, external private laboratories, or both, to identify if this accessibility may influence surveillance practices. Despite these limitations, the survey was carefully designed with input from senior vascular surgeons, pilot tested for clarity, and achieved reasonable geographic representation across both countries.

## 5 Conclusion

There is substantial practice variation in post‐endovascular therapy surveillance among Australian and New Zealand vascular surgeons that is independent of surgeon experience, practice setting (public vs. private), or geographic location. While most employ DUS surveillance, the frequency, duration, and intervention thresholds differ markedly. The high level of clinical equipoise (82%) and interest in participating in future research (87% expressing interest or potential interest) strongly supports the need for the planned SURVEIL randomized controlled trial. These findings provide valuable baseline data and demonstrate the clinical equipoise necessary to justify a clinical trial comparing surveillance strategies. Future studies should focus on identifying which patients benefit most from intensive surveillance and determining the optimal surveillance protocols that balance clinical outcomes with resource utilization.

## Conflicts of Interest

R.L.V. is a consultant to Medtronic, Abbott, Philips, W.L. Gore, BD, Boston Scientific, R3 Medical, Vesteck, Nectero, Intervene, Surmodics, Endospan, Cook Medical, Concept Medical, and Inari. He declares equity in Provisio Medical and Vesteck. S.D.T. is a consultant for BD, Abbott, and Medtronic. The other authors declare no conflicts of interest.

## Supporting information


**Data S1:** ans70329‐sup‐0001‐Supinfo.pdf.

## Data Availability

The data that support the findings of this study are available on request from the corresponding author. The data are not publicly available due to privacy or ethical restrictions.
