# Supplementary material for: Duplex Ultrasound Surveillance After Endovascular Therapy for Peripheral Artery Disease: An Australian and New Zealand Study
Source: ANZ J Surg. 2025 Sep 25;96(1-2):164–9. doi: 10.1111/ans.70329 (PMC12953731; doi:10.1111/ans.70329)
Supplement: Supplementary file 1 — Data S1: ans70329‐sup‐0001‐Supinfo.pdf. [file ANS-96-164-s001.pdf]

Intro

There are currently no RCTs comparing surveillance strategies following endovascular interventions of the lower limb. Subsequently, there is no clear guidance in the international guidelines (GVG, SVS, ESVS).

This survey aims to identify the current follow-up practices of vascular surgeons in Australia and Aotearoa New Zealand. In particular, to review the practice of duplex ultrasound surveillance after endovascular intervention for the lower limb. These questions specifically refer to the management of chronic peripheral arterial disease.

This survey should take no more than 10 minutes. Thank you for participating.

Demographics

How long have you been working as a consultant vascular surgeon (FRACS or equivalent)

- ○ <5 years
- ○ 5-9 years
- ○ 10-20 years
- ○ ≥20 years

What kind of hospital do you principally work in?

- ○ Teaching hospital
- ○ Metropolitan non-teaching hospital
- ○ Regional non-teaching hospital

What region of Australia / Aotearoa New Zealand do you principally work in?

- ○ Aotearoa New Zealand
- ○ Australian Capital Territory
- ○ New South Wales
- ○ Northern Territory
- ○ Queensland
- ○ South Australia
- ○ Tasmania
- ○ Victoria
- ○ Western Australia

What portion of public and private surgical work do you perform?

- ○ Chiefly public work (little or no private work)
- ○ Chiefly private work (little or no public work)
- ○ A mix of public and private work

Do you perform endovascular interventions (angioplasty, stenting, etc.) for lower limb peripheral arterial disease?

- ○ Yes
- ○ No

In what kind of room do perform endovascular interventions? (Select all that apply)

- ☐ Hybrid theatre
- ☐ Interventional / Radiology suite
- ☐ Operative theatre with mobile X-ray

What investigations do you have access to? (Select all that apply)

- ☐ Ankle Brachial Pressure Index (ABI)
- ☐ Toe Brachial Pressure Index (TBI)
- ☐ Duplex Ultrasound Scan (DUS)
- ☐ Computed Tomography Angiography (CTA)

☐ [ ] Other (Please specify)

Clinician's Practice

The following questions refer to routine post-procedural follow-up. This refers to a clinical review and/or investigation that is done at one or more timepoints following endovascular intervention, regardless of whether a patient is symptomatic from their arterial disease.

Following lower limb endovascular intervention, do you perform an initial post-procedure Duplex Ultrasound Scan (e.g. at 4-6 weeks post procedure)?

- ○ Yes
- ○ No

Do you perform routine post-procedural follow up (of any modality, e.g. clinical, ABI, DUS, CTA, etc.) after lower limb endovascular intervention, beyond the initial post-procedural review?

- ○ Yes
- ○ No

What modalities do you use for routine post-procedural surveillance? (Select all that apply)

- [ ] Clinical review
- [ ] ABI
- [ ] TBI
- [ ] DUS
- [ ] CTA
- [ ] ________ Other (Please specify)

Do you perform routine post-procedural duplex ultrasound surveillance after lower limb endovascular intervention (i.e. beyond the initial post-procedural review)?

- ○ Yes
- ○ No

Please select the lower limb arterial segments that you would routinely perform post-intervention duplex ultrasound surveillance. (Select all that apply)

- [ ] Aortoiliac Disease
- [ ] Femoropopliteal Disease
- [ ] Infrapopliteal Disease

Which patients (from an indication perspective) do you routinely surveil post-intervention? (Select all that apply)

- [ ] Intermittent claudication
- [ ] Rest pain (CLTI)
- [ ] Tissue loss (CLTI)

Which patients (from an intervention technology perspective) do you routinely surveil post-intervention? (Select all that apply)

- [ ] Angioplasty (including drug-coated balloon angioplasty)
- [ ] Stenting
- [ ] Atherectomy
- [ ] ______ Other (please specify)

How long do you surveil patients following intervention?

- ○ 3 months
- ○ 6 months
- ○ 12 months
- ○ 2 years
- ○ 5 years
- ○ Lifelong
- ○ ______ Other

What regularity of surveillance do you perform post-intervention? (e.g. every 3 months, every 6 months)

- ◯ 3 months
- ◯ 6 months
- ◯ 12 months
- ◯ Varies / depends on patient or lesion factors

In which circumstances would you consider reintervention for asymptomatic restenosis identified on routine post-intervention surveillance, in the aortoiliac segment? (Select all that apply)

- [ ] Moderate (50-75%) restenosis, regardless of ABI
- [ ] Moderate (50-75%) restenosis, with an ABI reduction of ≥0.1
- [ ] Severe (>75%) restenosis, regardless of ABI
- [ ] Severe (>75%) restenosis, with an ABI reduction of ≥0.1
- [ ] Not unless symptomatic

In which circumstances would you consider reintervention for asymptomatic restenosis identified on routine post-intervention surveillance, in the femoropopliteal segment? (Select all that apply)

- [ ] Moderate (50-75%) restenosis, regardless of ABI
- [ ] Moderate (50-75%) restenosis, with an ABI reduction of ≥0.1
- [ ] Severe (>75%) restenosis, regardless of ABI

- [ ] Severe (>75%) restenosis, with an ABI reduction of ≥0.1
- [ ] Not unless symptomatic

In which circumstances would you consider reintervention for asymptomatic restenosis identified on routine post-intervention surveillance, in the infrapopliteal segment? (Select all that apply)

- [ ] Moderate (50-75%) restenosis, regardless of ABI
- [ ] Moderate (50-75%) restenosis, with an ABI reduction of ≥0.1
- [ ] Severe (>75%) restenosis, regardless of ABI
- [ ] Severe (>75%) restenosis, with an ABI reduction of ≥0.1
- [ ] Not unless symptomatic

In which patients (from an indication perspective) would you consider reintervention for an asymptomatic restenosis? (Select all that apply)

- [ ] Intermittent claudication
- [ ] Rest pain (CLTI)
- [ ] Tissue loss (CLTI)

In which patients (in regard to the technology used in the initial procedure) would you consider reintervention for an asymptomatic restenosis? (Select all that apply)

- [ ] Angioplasty (including drug-coated balloon angioplasty)

- [ ] Stenting
- [ ] Atherectomy
- [ ] Other (please specify)

RCT

Thank you for your input so far, it's really appreciated.

We are in the planning stage of running an Australia and Aotearoa New Zealand based, multicentre, randomised trial comparing "clinical" (physical examination and ABI) vs "duplex" (clinical review with arterial duplex ultrasound) follow up programs following endovascular intervention in the femoropopliteal segment for peripheral arterial disease. This trial will compare clinical AND economic outcomes of the surveillance strategies.

The following questions are to identify clinicians' interest and capacity to participate in such a trial.

Do you feel there is equipoise in this question? i.e. that there is reasonable uncertainty in the relative risks and benefits of these surveillance strategies, such that it is reasonable to compare these approaches in a clinical trial?

- ○ Yes
- ○ No
- ○ Not sure

If "no", or "not sure", we would really like to hear your perspective on this research question (optional)

Would you be interested in participating in a trial comparing these surveillance strategies?

- ○ Yes
- ○ No
- ○ Maybe

Do you have the capacity to perform clinical reviews, ABI measurements and vascular ultrasounds in your unit?

- ○ Yes
- ○ No

In a 1:1 comparison, an enrolled patient has 50% chance of receiving regular duplex ultrasound surveillance. Would this

influence your willingness to enrol patients into a trial?

- ○ Yes
- ○ No
- ○ [ ] Not sure (please comment)

If this trial were to be randomised 2:1 (i.e. with 2 subjects in the "duplex" group for every 1 patient in the "clinical" group), would you be more willing to enrol patients into such a trial?

- ○ Yes
- ○ No
- ○ [ ] Not sure (please comment)

If you have any concerns or feedback about the proposed trial, we would appreciate your input (optional)

If you are interested in participating or learning more, could you please include your name, clinical unit and contact email address. The trial investigators will be in contact with you to provide further information regarding the proposed study.

- ☐ Name
- ☐ Clinical Unit
- ☐ Email address

Powered by Qualtrics
